# Evaluation of Health Care Professionals’ Knowledge, Attitudes, and Practice to Prevent the Pandemic Spread of COVID-19: A Questionnaire-Based Cross-Sectional Study from Abha, Saudi Arabia

**DOI:** 10.3390/healthcare11040509

**Published:** 2023-02-09

**Authors:** Moteb Khobrani, Rajalakshimi Vasudevan, Geetha Kandasamy, Jawaher A. Gramish, Kousalya Prabahar, Premalatha Paulsamy

**Affiliations:** 1 Department of Clinical Pharmacy, College of Pharmacy, King Khalid University, Abha 62529, Saudi Arabia; 2 Department of Pharmacology, College of Pharmacy, King Khalid University, Abha 62529, Saudi Arabia; 3 Department of Pharmacy Practice, College of Pharmacy, King Saud Bin Abdulaziz University for Health Sciences, Riyadh 14611, Saudi Arabia; 4 Clinical Pharmacy, Pharmaceutical Care Services, King Abdulaziz Medical City, National Guard Health Affairs, Riyadh 2915, Saudi Arabia; 5 Department of Pharmacy Practice, Faculty of Pharmacy, University of Tabuk, Tabuk 71491, Saudi Arabia; 6 Department of Nursing, College of Nursing, King Khalid University, Abha 62529, Saudi Arabia

**Keywords:** COVID-19, knowledge, attitude, practice, questionnaire, healthcare professionals

## Abstract

Background: Pharmacists and other healthcare professionals (HCPs) are at risk of transmitting the lethal COVID-19 virus globally and increasing its prevalence. Aim: The aim of this study was to assess knowledge, attitudes, and practices (KAP) about coronavirus disease 2019 (COVID-19) among HCPs in the Asir region for the first time. Methodology: A cross-sectional analysis with 491 healthcare professionals was tested using a pre-tested questionnaire in a tertiary care facility. The association between research variables and questions was determined using Chi-square tests and Kruskal–Wallis tests. Results: Pharmacists and other HCPs exhibited good knowledge, a positive attitude, and a negative practice pattern regarding COVID-19. There was a strong association between knowledge and attitude (correlation coefficient: 0.17; *p* < 0.001). However, healthcare practitioners had a suboptimal practice score of 2.09 ± 0.62 regarding COVID-19. Conclusion: This study discovered that despite relatively insufficient practices for adherence to recommended techniques regarding COVID-19 prevention during the outbreak, pharmacists and other healthcare professionals have a high level of awareness and a positive attitude towards COVID-19 as a medical condition. There is a need for more involved HCPs, improved COVID-19 management training, and approaches to make healthcare providers feel less anxious.

## 1. Introduction

Coronaviruses, which are transmitted through the air, produce infections ranging from the common cold to severe acute respiratory syndrome, and they impact people from all walks of life. Birds and animals have been observed to spread the virus in the past, with humans being particularly vulnerable to virus infection and transmission [1,2]. The novel coronavirus disease (COVID-19), which originated in China, has easily crossed borders, infecting people worldwide [3,4]. COVID-19 is caused by a virus known as 2019-nCoV, which was later renamed coronavirus 2 syndrome by the ICTV (International Committee on Taxonomy of Viruses) (SARS-CoV-2) [5,6].

SARS-CoV-2 appears to have originated in bats, and the first cases were reported in Wuhan, Hubei Province, China, indicating an animal-to-human transmission from a live animal market. The virus then expanded outside of Wuhan and ultimately to the rest of the world by human transmission [7,8]. On 2 March 2020, the first COVID-19 case in Saudi Arabia was discovered in a passenger returning from Iran [9]. As of 27 March 2020, the country had 1104 cases with three deaths. So far in Saudi Arabia, most patients appear to be returning tourists and their immediate contacts. At the time of writing, 113,356,584 people in 215 countries have been diagnosed with the disease, with an estimated 86,232,768 recoveries and 2,465,886 fatalities, for a 6.97 percent total fatality rate. There are cumulatively 374,691 cases in the Kingdom of Saudi Arabia (KSA), with 365,745 (98%) recoveries and 6457 (2%) deaths. With the rise in the prevalence of coronavirus, healthcare workers are in danger of contracting and transferring the deadly illness [10,11,12].

The World Health Organization (WHO) classified coronavirus illness as a pandemic on 11 March 2020. Apart from the strain of extended work hours, physical and psychological stress, burnout, and exhaustion, the highly infectious SARS-CoV-2 virus is an external healthcare hazard [13,14,15]. By the end of January, the WHO and the CDC (Centers for Disease Control and Prevention) had produced guidance for healthcare professionals (HCPs) on preventing and managing COVID-19. The WHO also hosted several online COVID-19 training sessions and resources in various languages to help strengthen preventive initiatives, such as increasing awareness and training HCPs [16,17]. In this light, the COVID-19 outbreak gives a unique chance to investigate the level of awareness and expectations of healthcare providers during this public health crisis.

Healthcare workers have an essential role in lowering morbidity and death, but yet they are directly exposed to patients and causal factors. For example, during the first COVID-19 outbreak in China [18], preventing nosocomial infections and protecting healthcare workers were significant problems for the healthcare system. If they do not have enough information and comprehension of the disease or do not take sufficient precautions, healthcare personnel are at significant risk from infection by patients [19]. Preventing cross-infection from patients while providing care can be accomplished if healthcare practitioners, such as doctors, pharmacists, nurses, and other medical professionals, have enough awareness, a positive mindset, and knowledge of best practices addressing COVID-19. Furthermore, healthcare teams’ more robust preventative policies and risk management are critical for a constructive reaction to new emerging threats [11,12,20,21].

Comprehensive information promotes a positive attitude and productive work activities, lowering the risk of infection. The awareness, perceptions, and practices of health professionals about COVID-19 have impacts on their adherence to control measures. As a result, in order to have proper safety measures, it is vital to evaluate medical practitioners’ competencies as well as the factors that influence their attitudes and practices [22].

The risk of contamination and cross-infection is higher for healthcare personnel. Frontline healthcare workers such as doctors, nurses, and pharmacists have been significantly impacted by the COVID-19 pandemic. The pandemic has killed hundreds of healthcare professionals. There is always a chance of a second wave, as with every infectious disease, in which the number of cases rises once more after a brief fall. According to a detailed assessment of available data, pandemic events throughout history, such as the Spanish flu, have occurred in waves, where subsequent waves are associated with increased mortality rates. However, due to the less severe nature of more recent outbreaks such as SARS and MERS in comparison to COVID-19, the second wave of such infections have been avoided [23,24,25]. When it comes to the application of protective limitations and protocols, social distancing, wearing of masks in public places, and practicing hand hygiene, those who do not completely comprehend the need for preventative measures are always seen as a liability. Medical personnel are consequently under increased strain.

The chance of infection is decreased by a positive attitude, good work practices, and comprehensive knowledge. Healthcare professionals’ (HCPs’) knowledge, attitudes, and practices (KAP) regarding COVID-19 have an impact on how well they follow control programs. To stop the virus’s transmission and ensure the effectiveness of the overall COVID-19 response, healthcare professionals knowledge, attitude, and practice are essential. The most recent research paints a contrasting image of HCPs’ KAP concerning COVID-19. Some research [26,27,28] has shown that HCPs follow safer infection, prevention, and control methods if recommended by international and local health authorities. On the other side, a study in Ethiopia found that HCPs had poor COVID-19 prevention practices, while a study in Nepal found that nearly half of HCPs had unfavorable views on COVID-19. Saudi Arabian healthcare professionals in a prior study on KAP were shown to have a solid comprehension of COVID-19. However, nurses who worked in critical care units made up the majority of the study’s participants (75%). Therefore, general healthcare workers still need to be familiar with the appropriate KAP. Additionally, it is crucial to comprehend the specialization of various classes of healthcare providers and pinpoint the variables influencing their attitudes and practices in order to promote proper practices and protection [29,30,31].

Therefore, the objective of this study was to assess the knowledge, attitudes, and practices of healthcare workers about COVID-19 infections, which could benefit containment efforts. The results of this study will assist us in identifying potential problems and formulating corrective actions to prevent additional outbreaks in healthcare facilities and the general population.

## 2. Methodology

### 2.1. Design of the Research

During the first week of November 2020, a prospective, web-based cross-sectional study was performed utilizing a survey questionnaire to acquire responses from healthcare practitioners.

### 2.2. Participants

The interviews were performed in Asir hospital in Saudi Arabia over the course of two months (November 2020–December 2020). The current study’s targeted population included all healthcare personnel employed in an Asir region tertiary care institution. In this study, healthcare workers such as physicians, pharmacists, nurses, and other paramedical personnel were considered eligible. The objectives and outcomes of the study were explained to all participants, and those who consented to sign the consent form were enrolled. A total of 491 persons participated in the study.

### 2.3. Study Tool

A standardized, self-administered questionnaire was used to collect data. This poll was created using a validated concern scale previously used in a study evaluating HCPs’ concerns about COVID-19. In addition, the WHO course materials on emerging respiratory viruses, including COVID-19, were used to create a 25-item survey instrument [32,33,34], which addressed the domains of HCP characteristics, awareness, information sources, knowledge, and perception linked to COVID-19.

There were four sections to the standardized, self-administered questionnaire. The first section contained demographic data about the respondents—age, gender, and occupation (3 items). The second section determined where respondents got their COVID-19 knowledge (media, webinars, journals, internet, family and friends—5 statements/5-point Likert scale). The next section tested healthcare workers’ understandings of COVID-19 by asking them to answer “Yes” or “No” to each set of questions. The final section examined respondents’ attitudes toward COVID-19, with their responses rated on a five-point Likert scale of agreement/disagreement. The survey instrument took about 3 min to complete on average. Respondents were given enough time overall to read, comprehend, and answer all of the questions.

The survey instrument used in this study tested HCPs’ knowledge of COVID-19’s nature, etiology, symptoms, risk group, consequences, and source of transmission, as well as its prevention and treatment. Knowledge scores varied from 0 to 12, with 8 as the cutoff for low versus good knowledge. Attitude was evaluated using an 8-item questionnaire with responses recorded on a 5-point Likert scale. Strongly agree received a score of 1, agree received a score of 2, undecided received a score of 3, disagree received a score of 4, and strongly disagree received a score of 5. A good attitude was defined as a mean score of 2, whereas a negative attitude was described as a score more than 2 and less than or equal to 5.

Practices of respondents towards COVID-19 was used to collect data on methods of prevention and control of COVID-19. Seven questions measured practices of HCPs towards COVID-19. The average score was measured and used as a cutoff for good, average, and insufficient practice on the practice questionnaire. A mean score of ≤2 was considered good practice, while a score of 2–3 was regarded as insufficient practice, and a score of 3–5 was considered poor practice.

### 2.4. Statistics

Data were coded, validated, and analyzed using the Statistical Package for the Social Sciences (SPSS), version 21. Descriptive analysis was applied to calculate the frequencies and proportions. The Pearson Chi-square test was used to check whether the respondents’ answers deviated from the expected correct answer. A *p*-value greater than 0.05 signaled no statistically significant departure from the correct answer. It was also used to assess differences (e.g., between males and females) in their responses concerning particular practices. To identify the association between demographic characteristics and knowledge, attitude, and practice of COVID-19, the Kruskal–Wallis test was applied. A *p*-value less than 0.05 indicated a significant difference between groups with respect to their responses to these variables.

### 2.5. Ethical Approval

The study was approved by research committee ECM#2020-239-HAPO-06-B-001 and conducted in a tertiary care hospital. Furthermore, written consent was obtained from the respondents prior to participation in the study.

## 3. Results

### 3.1. Socio-Demographic Characteristics

**Figure 1:**
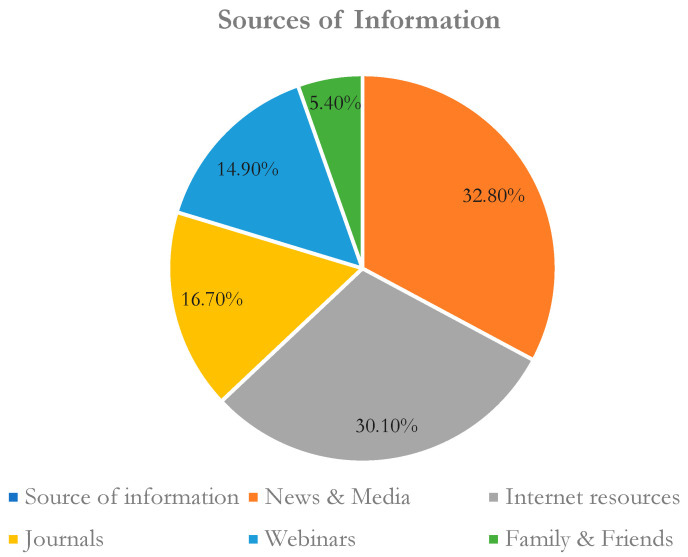
Sources of information related to COVID-19 reported by healthcare workers.

**Figure 2:**
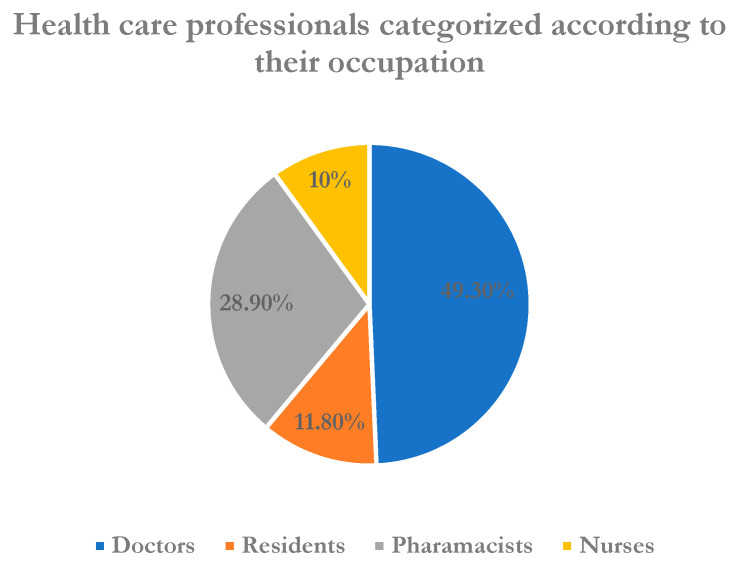
Healthcare professionals categorized according to their occupation (N = 491).

About 491 healthcare workers responded to the questionnaire, yielding a response rate of 95.71%. The majority was male (55.6%) and belonged to major healthcare professions, with physicians and pharmacists comprising the most respondents (49.3% and 28.9%, respectively). Most of them (n = 396) were less than 30 years and had just started their job with minimum experience, with about 75% having less than three years of experience. More than one-third of the participants were categorized as frontline healthcare professionals. Respondents’ characteristics are shown in Table 1. The main source of information reported by participants was the Internet, as shown in Figure 1. In addition, the participants’ occupational distribution is shown in Figure 2.

### 3.2. Knowledge of HCPs about COVID-19

Table 2 describes the status of COVID-19 knowledge among HCPs. About 412 (84%) of respondents showed good knowledge, while 79 (16%) of patients had poor knowledge of COVID-19. Respondent awareness of COVID’s origin, since the Chi-square value was 0.059, indicated departure from the expected correct answer, namely, that it originated in bats. The study showed that average knowledge was more apparent in response to questions regarding the transmission, treatment, availability of critical care, and the consequences of COVID-19 in children, with the rate of correct responses being 71.89%, 72.3%, and 70.3%, respectively. The mean knowledge score of healthcare workers was 9.17 ± 1.48.

### 3.3. HCPs’ Attitudes towards COVID-19

About 369 (75.15 percent) of 491 respondents displayed a positive attitude to COVID-19, while 122 (24.85 percent) respondents showed a negative attitude to COVID-19. On average, respondents displayed negative attitudes towards the condition and actions that might be taken when asked about intensive care to be given to diagnosed patients (2.26 ± 1.17), and there was also less COVID-19 anxiety (2.28 ± 0.99). Conversely, when asked about the use of gowns, gloves, and other safety measures when working with COVID-19 patients, most participants replied positively (1.44 ± 0.79). In addition, different groups of professionals and those with different levels of experience diverged in their level of agreement on three of the questions (2, 3, and 8). Table 3 summarizes the findings.

### 3.4. Mean Score of Knowledge, Attitude, and Practice (KAP)

The association of demographic characteristics with mean knowledge and attitude questions is expressed in Table 4. Among the demographic variables, profession and category of respondents were significantly associated with mean knowledge and attitude scores. Level of experience just missed statistical significance. These results align with those of Table 3 on attitude. Frontline healthcare professionals showed more knowledge (9.28 vs. 8.98, *p* = 0.05) and positive attitudes (1.68 vs. 1.99, *p* = 0.005) towards COVID-19 compared to their non-frontline counterparts. Similarly, it was also revealed that doctors have more knowledge and a positive attitude compared to those who are less involved in the field. A Spearman correlation test revealed a significant positive relationship between knowledge and attitude of healthcare workers regarding COVID-19 (r = 0.17, *p* < 0.001).

### 3.5. HCPs’ Practices towards COVID-19

On average, for COVID-19, there was an inadequate practice score of 2.09 ± 0.62 among healthcare practitioners. Approximately 245 (49.89 percent) of 491 respondents showed good practices towards COVID-19, while 246 (50.1 percent) respondents were shown to be inadequate towards COVID-19. Inadequate practices were observed when HCPs were asked about diagnostic centers (2.17 ± 0.9) and five steps (2.36 ± 1.07) of the hand washing technique. In addition, insufficient practices were observed for adherence to recommended techniques such as nasopharyngeal swabbing (2.41 ± 1.19) and procedures (2.03 ± 1.03) for hand washing. The categories of professionals and males versus females were noted to differ in these two areas. Conversely, most participants displayed good experience (1.92 ± 0.98) when asked about safety precautions such as quarantine with the family when dealing with COVID-19 patients. Table 5 summarizes the findings.

## 4. Discussion

### 4.1. Summary and Comparisons

COVID-19 is presently a global topic of discussion among the media and the public, and especially HCPs and patients. This pervasiveness raises a significant recurring concern about how we handle knowledge to assist frontline healthcare workers in times of public health crisis. The transition towards a COVID-19 environment raises pressure on all, including health officials and the health system. For this reason, during a global outbreak, we examined the awareness and attitudes of HCPs towards the prevention and control of COVID-19.

The participants in this study exhibited good awareness and a positive attitude towards the novel coronavirus (COVID-19). This overall assessment is similar to that of a study conducted by Kara et al., which indicated that healthcare workers with good knowledge regarding COVID-19 also showed good attitudes and engaged in good practices. Most of our respondents in the study were aware of the coronavirus from news, media, and Internet sources. This detail was confirmed by a study that showed technologies are a critical source of awareness of such viruses by the participants [32]. Healthcare workers are now more reliant than ever on Internet technologies to gain knowledge of novel diseases such as SARS-CoV-2 [35]. Most of the educational materials on COVID-19 are posted online by the Ministry of Health, which may have urged the HCPs to use Internet technology to access those documents. A key factor in HCWs’ preparedness and response is trust that transparent knowledge about the emerging COVID-19 infection derives from authentic sources. Therefore, healthcare professionals should place the focus on skills for the retrieval of information on the Internet and its assessment.

Moreover, knowledge regarding COVID-19 symptoms, incubation period, treatment, and preventing the spread of the disease was assessed. The observed results may be due to health authorities’ focus on these issues in their training programs. The current findings are consistent with other studies showing HCPs’ positive responses to prevention of disease when dealing with SARS-CoV-2 [12,36,37].

In terms of awareness of symptoms, however, the outcome of the current study is not in line with one conducted by Bhagavathula et al. in 2020 [34] to assess healthcare workers’ knowledge of COVID-19. Respondents in that study displayed poor knowledge when questioned about disease prevention and symptoms. One explanation for the disparity in findings can be due to educational campaigns in Saudi Arabia having concentrated more on the signs and symptoms of COVID-19, which might be strengthening people’s awareness in this area.

Approximately one-third (29.7%) of healthcare professionals were not well aware of COVID-19 preventive strategies for younger adults and children. About 27.6 percent of respondents answered incorrectly about the supportive care and specific treatment for COVID-19 available in their zone. In contrast to less experienced peers, experienced staff correctly answered this question. Therefore, this aspect of COVID-19 must be emphasized so that HCPs can play their part by educating individuals to address the threat to global public health of such a novel virus.

The current study observed that the mean attitude score was in the positive range. The healthcare workers’ most optimistic approach was to use protective equipment when interacting with COVID-19 patients (1.44 ± 0.79). This result is in line with another study showing healthcare workers’ positive reactions to wearing goggles and gloves while coping with healthcare-related infections [38]. Our study also observed that relative to non-frontline HCPs, frontline healthcare professionals replied positively to most of the attitude-related questions (*p* < 0.05). A different attitude was found when respondents reacted negatively to the prioritization of intensive care and emergency treatment. This outcome is consistent with another study conducted by Huynh et al. noting the positive attitude of healthcare staff towards active involvement in the hospital control program [20]. No significant attitudinal variations between male and female healthcare professionals in our study were identified. For example, the pandemic’s emergency centeredness, the actual medical system, and the paucity of resources compelled medical practitioners to make pragmatic, sometimes ethically changing decisions out of necessity [11].

The findings align with other studies that have reported positive physician attitudes among all health workers [39,40]. The statistically significant correlation between healthcare workers’ awareness and attitudes reaffirms the positive relationship between COVID-19 knowledge and attitude. Thus, HCPs with a more optimistic attitude toward COVID-19 are encouraged to seek more information about the disease and improve their knowledge base. Nevertheless, future research must be undertaken to fully understand the pattern of information flow and HCPs’ expressed attitudes. Our study additionally shows that professional category and level of experience are significant factors in readiness to participate in hospital infection control and information sharing with peers.

Conversely, there was an inadequate practice score of 2.09 ± 0.62 among healthcare practitioners towards COVID-19. The practice of hand hygiene is essential. It is very beneficial for infection prevention, and adequate hand hygiene practice is also encouraged by governments and many other stakeholders. Tamang et al. also stated that one of the critical barriers to disease transmission is hand washing [41]. We asked participants in this survey if they believed in taking all five WHO-recommended hand washing steps; about 57 percent of the HCPs either strongly agreed or agreed. Seventy percent of the participants responded positively when asked whether they had attended hand hygiene training, but the remaining participants reported that they were neutral, disagreed, or strongly disagreed. These findings indicate that the majority of our participants find hand hygiene procedures warranted. However, we still think that hand hygiene is the most overlooked operation, and HCPs do not typically follow all the moments and steps. Of note, we found a statistically significant difference between respondents from different professions on hand washing, a finding worth exploring.

### 4.2. Study Limitations

A web-based survey was conducted to identify the knowledge, attitudes, and practices (KAP) of healthcare professionals operating in the nation’s numerous hospitals where they have full Internet service access. A physical assessment at these facilities was not possible. Furthermore, as our research was performed primarily via an online self-administered questionnaire, we could not observe the pattern of personal protective equipment (PPE) use and had to rely on self-reported evaluation for that. Another limitation was that our study did not include laboratory technicians and pharmaceutical employees. Despite all these constraints, the KAP survey on COVID-19 among HCPs in the Asir area maintained representativeness for the professionals we assessed.

### 4.3. Implications of Research

Healthcare professionals’ awareness plays a vital role in reducing morbidity and mortality, as they are directly exposed to patients and causative agents. Comprehensive knowledge encourages a positive approach and constructive activities at work, which reduces the risk of COVID-19 infection. The study recommends implementing educational and professional campaigns to increase healthcare professionals’ awareness, which would also positively affect their COVID-19 work practices.

## 5. Conclusions

Healthcare professionals in the Asir region of Saudi Arabia showed adequate knowledge and positive attitudes towards COVID-19 and mitigation steps. Still, there is a need for development in certain areas, such as effective treatment of such pandemic diseases. We found a significant correlation between COVID-19 awareness and positive attitudes among healthcare professionals. The attitudes of healthcare professionals are directly linked to their expertise. Thus, despite improved awareness and attitudes, more deliberate practice efforts are required. Training in preventive and safety measures is also necessary to encourage best working practices during the pandemic response to COVID-19. Our study recommends further implementation of educational and professional campaigns to increase HCP awareness, which would also have a positive impact on their COVID-19 working practices.

## Figures and Tables

**Table 1:** Distribution of healthcare professionals according to their socio-demographic characteristics.

Socio-Demographic Characteristics	Health Care Professionals	
	n (491)	%
Age		
Less than 30 years	396	80.7
30–39 years	69	14.1
40–49 years	19	3.9
More than 50 years	7	1.4
Sex		
Male	273	55.6
Female	218	44.4
Occupation		
Doctors	242	49.3
Residents	58	11.8
Pharmacists	142	28.9
Nurses	49	10
Category of healthcare professionals		
Frontline HCPs	180	36.7
Non-frontline HCPs	311	63.1
Experience		
Less than 3 years	371	75.6
3–6 years	51	10.4
7–10 years	21	4.3
More than 10 years	48	9.8
Attended lectures/discussions about novel COVID-19		
Yes	381	77.2
No	110	22.8

**Table 2:** Knowledge of HCPs about COVID-19.

Questions Related to Knowledge of COVID-19	Correct Response	True n (%)	False n (%)	Chi Square Test (X^2^)
Coronavirus is a viral disease caused by a beta coronavirus thought to be originated from bats.	True	407 (82.89)	84 (17.1)	0.059
Incubation period for virus is 2–14 days.	True	460 (93.68)	31 (6.31)	0.329
Fever, dry cough, shortness of breath, and tiredness are hallmark symptoms of COVID-19.	True	471 (95.9)	20 (5.1)	0.589
Not all persons with COVID-19 will develop severe cases. Only those who are elderly having chronic illnesses are more likely to be severe cases.	True	402 (81.8)	89 (18.1)	0.486
Majority of COVID-19 infective patient will not develop severe illness but elderly, patient having chronic illness, DM, COPD are likely to develop severe illness.	True	429 (87.37)	62 (12.6)	0.498
Persons with COVID-19 cannot transmit to others if fever and dry cough is not present.	False	138 (28.1)	353 (71.89)	0.432
Polymerase chain reaction (PCR) can be used to diagnose COVID-19.	True	406 (82.68)	85 (17.31)	0.048 *
Currently there is no treatment of COVID-19 infection, but early symptomatic and supportive treatment can help most patient recover from infection.	True	460 (93.68)	31 (6.3)	0.385
Supportive care and specific treatment for COVID-19 is available in Asir region.	True	355 (72.3)	136 (27.6)	0.447
Suspected COVID-19 patient should be sent to a quarantine center or home quarantine.	True	445 (90.6)	46 (9.4)	0.168
Wearing general medical masks can prevent one from acquiring infection by the COVID-19 virus.	True	416 (84.73)	75 (15.27)	0.416
It is not necessary for children and young adults to take measures to prevent the infection by the COVID-19 virus.	False	146 (29.7)	345 (70.3)	0.288

Note: * Chi-square test (*p* < 0.05) Knowledge score was evaluated by giving 1 for responding correctly and 0 for responding incorrectly. The scale ranged from 12 to 0. A score of less than 8 was considered poor knowledge, while a score of greater than 8 was considered good. The mean score for knowledge was 9.17 ± 1.48.

**Table 3:** HCPs’ attitudes towards COVID-19.

Questions Pertaining to Attitude	HCPs’ Responses N (%)					*p*-Value *			
	SA	A	N	D	SD	Age	Sex	Prof.	Exp.
Transmission of COVID-19 infection can be prevented by using universal precautions given by the CDC, WHO, etc. ^a^	224 (45.6)	190 (38.7)	58 (11.8)	12 (2.4)	7 (1.4)	0.655	0.902	0.148	0.443
The prevalence of COVID-19 can be reduced by the active participation of healthcare professionals in the hospital infection control program. ^b^	196 (39.9)	196 (39.9)	78 (15.8)	13 (2.6)	7 (1.4)	0.911	0.702	0.06	0.043
Any related information about COVID-19 should be disseminated among peers and other healthcare professionals. ^c^	206 (41.9)	177 (36)	82 (16.7)	14 (2.8)	12 (2.4)	0.870	0.770	0.045	0.294
Intensive and emergency treatment should be given to diagnosed patients. ^d^	142 (28.9)	152 (30.9)	113 (23)	58 (11.8)	26 (5.2)	0.549	0.116	0.257	0.995
Do you think quarantine of suspected COVID-19 cases for 14 days can reduce the spread of the infection? ^e^	266 (54.1)	137 (27.9)	51 (10.3)	29 (5.9)	8 (1.6)	0.350	0.855	0.334	0.437
Level of fear of COVID-19 is very high. ^f^	135 (27.4)	168 (34.2)	139 (28.3)	41 (8.3)	8 (1.6)	0.250	0.573	0.811	0.328
Gowns, gloves, mask, and goggles must be used when dealing with COVID-19 patients. ^g^	352 (71.6)	82 (16.7)	40 (8.1)	15 (3)	2 (0.1)	0.910	0.166	0.200	0.608
Regulation taken by the government is enough to combat disease. ^h^	207 (42.1)	151 (30.7)	78 (15.8)	42 (8.5)	13 (2.6)	0.593	0.382	0.012	0.006

Prof.—Profession; Exp—Experience; SA = strongly agree, A = agree, N = neutral, D = disagree, SD = strongly disagree. * Derived from Chi-square test. Note: Attitude was measured by defining 1 for SA, 2 for A, 3 for U, 4 for D, 5 for SD. A score of <2 was taken as a positive attitude and <2 a negative attitude. Mean attitude score was 1.88 ± 0.88. Mean attitude score ± S.D: ^a^ 1.71 ± 0.85; ^b^ 1.82 ± 0.87; ^c^ 1.85 ± 0.94; ^d^ 2.26 ± 1.17; ^e^ 1.69 ± 0.96; ^f^ 2.28 ± 0.99; ^g^ 1.44 ± 0.79; ^h^ 1.90 ± 0.09.

**Table 4:** Mean score of knowledge, attitude, and practice (KAP).

Characteristics	N	Knowledge Score (Mean ± S.D)	*p* Value	Attitude Score (Mean ± S.D)	*p* Value	Practice Score (Mean ± S.D)	*p* Value
Age							
Less than 30 years	396	9.15 ± 1.21	0.542	1.90 ± 0.45	0.544	2.09 ± 0.31	0.741
30–39 years	69	9.22 ± 1.35		1.87 ± 0.44		2.02 ± 0.21	
40–49 years	19	8.94 ± 1.62		1.90 ± 0.18		2.18 ± 0.45	
More than 50 years	7	9.57 ± 1.16		1.68 ± 0.34		1.89 ± 0.22	
Gender							
Male	273	9.22 ± 1.25	0.453	1.89 ± 0.12	0.892	2.06 ± 0.34	0.653
Female	218	9.05 ± 1.39		1.89 ± 0.19		2.10 ± 0.47	
Profession *							
Doctors	242	9.20 ± 1.16	0.044	1.85 ± 0.37	0.058	1.95 ± 0.19	0.052
Residents	58	9.05 ± 1.31		1.99 ± 0.25		2.02 ± 0.42	
Paramedics	142	9.15 ± 1.26		2.13 ± 0.31		2.07 ± 0.41	
Nurses	49	8.91 ±1.34		1.76 ± 0.41		1.83 ± 0.21	
Experience							
Less than 3 years	371	9.19 ± 1.23	0.064	1.88 ± 0.12	0.482	2.06 ± 0.23	0.851
3–6 years	51	9.14 ± 1.44		1.98 ± 0.31		2.08 ± 0.44	
7–10 years	21	8.57 ± 1.37		1.99 ± 0.27		2.14 ± 0.32	
More than 10 years	48	9.15 ± 1.21		1.89 ± 0.15		2.09 ± 0.21	
Category of respondents *							
Frontline HCPs	180	9.28 ± 1.19	0.054 *	1.68 ± 0.12	0.045 *	1.98 ± 0.14	0.051
Non-frontline HCPs	311	8.98 ± 1.26		1.99 ± 0.34		2.12 ± 0.17	

* Kruskal–Wallis Test (*p* < 0.05).

**Table 5:** Practice towards COVID-19.

Questions Pertaining to Practice	HCPs’ Responses N (%)					*p*-Value *			
	SA	A	N	D	SD	Age	Sex	Prof.	Exp.
Preventive measures against COVID-19 are adequately followed by healthcare professionals. ^1^	180 (36.6)	197 (40.1)	89 (18.1)	20 (4)	5 (1)	0.503	0.264	0.177	0.166
Do you think diagnostic centers are approachable and the results are accurate? ^2^	119 (24.2)	206 (41.9)	128 (26.1)	32 (6.5)	6 (1.2)	0.888	0.720	0.464	0.718
Do you think all the healthcare professionals follow all the five steps (WHO recommended) of the hand washing technique? ^3^	110 (22.4)	170 (34.6)	123 (25.6)	70 (14.2)	13 (2.6)	0.286	0.462	0.017*	0.481
Attended training on hand hygiene, wearing and removing facemask during COVID-19 pandemic. ^4^	184 (37.5)	163 (33.1)	98 (19.9)	33 (6.7)	13 (2.6)	0.582	0.052	0.152	0.067
Attended training on performing nasopharyngeal swab safely. ^5^	128 (26.1)	148 (30.1)	113 (23)	69 (14.1)	33 (6)	0.911	0.002*	0.188	0.879
Obtained knowledge about COVID-19 pandemic mainly from social websites of Saudi MOH, WHO, CDC. ^6^	206 (41.9)	189 (38.5)	69 (13.8)	22 (4)	5 (1)	0.303	0.255	0.083	0.746
During the outbreak, whether you maintained quarantine with family? ^7^	215 (43.8)	144 (29.3)	86 (17.5)	39 (7.9)	7 (1.4)	0.976	0.384	0.102	0.925

Prof.—Profession; Exp—Experience; SA = strongly agree, A = agree, N = neutral, D = disagree, SD = strongly disagree. * Derived from Chi-square test. Note: Practice was measured by defining 1 for SA, 2 for A, 3 for U, 4 for D, 5 for SD. A mean score of ≤2 was considered good practice, while a score of 2–3 was regarded as insufficient practice, and a score of 3–5 was considered poor practice. Mean practice score overall was 2.09 ± 0.62. Mean practice score ± S.D: 1.923 ± 0.875 ^1^; 2.17 ± 0.9 ^2^; 2.366 ± 1.067 ^3^; 2.03 ± 1.03 ^4^; 2.41 ± 1.19 ^5^; 1.80 ± 0.85 ^6^; 1.92 ± 0.98 ^7^.

## Data Availability

The datat used in the current study will be made available on request from Rajalakshimi Vasudevan; raja@kku.edu.sa.
